# Turn down your thermostats – A contribution to overcoming the European gas crisis? The example of Germany

**DOI:** 10.1016/j.heliyon.2024.e23974

**Published:** 2024-01-10

**Authors:** Evelyn Sperber, Ulrich Frey, Valentin Bertsch

**Affiliations:** a German Aerospace Center (DLR), Institute of Networked Energy Systems, Curiestr. 4, 70563, Stuttgart, Germany; b Ruhr-Universität Bochum, Chair of Energy Systems and Energy Economics, Universitätsstr. 150, 44801, Bochum, Germany

**Keywords:** Gas crisis, Temperature setpoint, Gas savings, Consumer bill, Reduced-order building model, Building characteristics

## Abstract

Europe's current gas crisis requires rapid government intervention to curtail natural gas consumption and mitigate expenses for consumers. This study aims to comprehensively assess the impact of adjusting thermostats, with single-family houses in Germany serving as a case study. A unique bottom-up approach for approximating gas consumption at the level of building archetypes reveals that decreasing temperature setpoints from 21 °C to 19 °C and 17 °C can save about 14 and 30 TWh/a of gas, respectively. This corresponds to 3–6 % of gas imports from Russia to Germany in 2020. The largest absolute savings can be realized in older and larger buildings. Additionally, our findings suggest that the adjustment of thermostats may decrease residential CO_2_ emissions by 3–6 Mt/a, achieved through a reduction of 2–4 °C in the setpoint. Therefore, the measure shows great promise regardless of the present crisis. From the consumer's perspective, a 1 °C temperature reduction can lead to a gas bill reduction of 4–9 %, contingent upon building type. Nevertheless, the cost burden associated with rising gas prices surpasses these savings. Residents of older buildings suffer more severe financial impacts than those in newer ones. Our research suggests that policymakers should consider implementing adjustments to residential thermostats. Furthermore, consumer financial assistance programs should factor in building type when designing relief mechanisms.

## Highlights

• Demonstrates bottom-up quantification of gas savings from thermostat adjustments.
• Saving potentials vary with building age, size and temperature settings.
• Up to 6 % of Russian gas imports can be cut by German single-family households.
• Monetary savings from thermostat adjustments do not outweigh gas price increases.
• Consumers should be relieved according to building type.

## 1 Introduction

### 1.1 Motivation

The Russian invasion of Ukraine in February 2022 has triggered an international energy crisis. The already tight energy markets have experienced a shock the likes of which have not been felt since the last oil crisis at the end of the 1970s. This external shock hit Europe and Germany in particular, which are heavily dependent on cheap gas from Russia – both as an energy carrier and as a resource, e.g., for the chemical industry. Before the war, about 40 % of natural gas imports to the European Union originated from Russia (including transits from Ukraine and Belarus) [1]. Significantly less gas has been flowing since mid-May 2022 through Nord Stream 1, the most important pipeline for Russian gas to the European Union, and finally stopped by the end of August 2022 [2]. This puts energy security at high risk. For Germany, Russian gas imports were as high as 55 % in 2020 [3]. In the course of the war, supply from Russia has been disrupted.

Markets have anticipated this shortfall and prices of natural gas have increased significantly. Meanwhile, at Europe's most important trading point “Title Transfer Facility” (TTF), futures for winter 2022/2023 temporarily were at over 300 €/MWh – a twenty-fold increase from 2020 levels [4]. The price situation on the gas wholesale markets has eased somewhat since then. However, prices for the coming months are still in the range of double to quadruple of pre-crisis levels. Furthermore, this gas price shock has translated into rising electricity wholesale prices in many European markets [5]. Retail prices are also increasing, although with delay. In Germany, the consumer price index for natural gas in the winter months 2022/2023 was more than three times the previous year's levels [6].

Thus, pressure mounted rapidly for politicians to act. Following its ”Save gas for a safe winter” plan [7], the European Union succeeded in achieving its voluntary reduction target for gas consumption of 15 % from August 1, 2022 to March 31, 2023. In combination with filled gas storage facilities (which in 2022 were still significantly dependent on Russian gas), security of supply could thus be maintained in the winter of 2022/2023. However, a gas shortage is considered a serious risk by the European Commission for the following winter(s), too, as a result of the significant decrease in imports of Russian gas in the course of 2022. In addition, the situation is exacerbated by several factors such as the recovery of demand for LNG in Asia and poor weather conditions related to the operation of hydro and nuclear power plants. Consequently, the savings target of 15 % natural gas has been extended for another year until the end of March 2024 [8].

Several measures have been discussed or already been implemented to comply with this target [7,[9], [10], [11]]. These proposed measures apply to all sectors of the energy system. On the one hand, this includes short-term measures like fuel switching in the industry and power generation as well as savings in households and the commercial sector. On the other hand, more long-term, structural measures are the decarbonization of the building sector as well as the massive expansion of renewable energies and hydrogen production. It is also proposed to diversify natural gas imports, for example by increasing LNG import capacity [7,[9], [10], [11]].

**Fig. 1:**
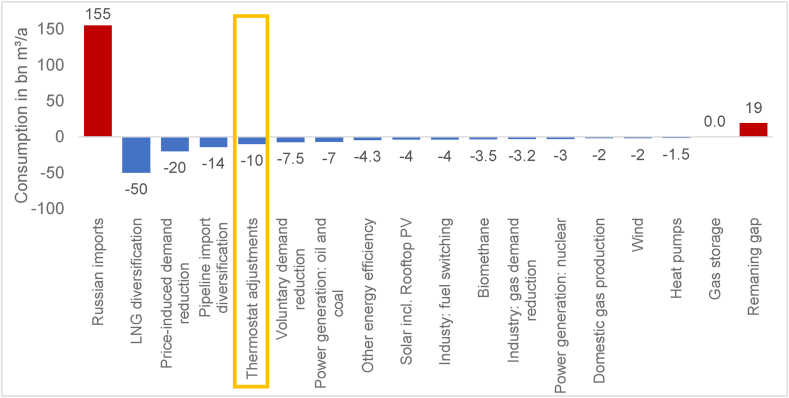
Proposed gas saving measures by the European commission including estimation of effects, own illustration based on [7].

Fig. 1 summarizes the European Commission's assessment of different measures. Taken together, these measures come close to closing the gas supply gap caused by shortfalls from Russia. While this provides an indication of promising measures, a more in-depth analysis of individual measures is required to both robustly determine their effectiveness and assess potential societal impacts. Ultimately, this is crucial for designing effective and equitable policy instruments.

This paper therefore analyses (voluntary) energy savings, in particular thermostat adjustments in residential buildings (Fig. 1, yellow marking). Along with gas import diversification and price-induced demand reduction, this is one of the most promising measures. The high relevance of the building heating sector from the perspective of the energy system and the individual consumer adds to its importance.

In Europe, residential heating accounts for about a third of final energy consumption [12]. In turn, space heating is highly dependent on natural gas – the fuel covers about half of the European Union's primary energy demand for space heating [12]. As a consequence, household consumption expenditures are also highly dependent on gas prices. German households, for instance, used to spend about 7 % of their income for energy in 2020. As soon as March 2022, however, this share increased to 9.4 % and could well be much higher in future due to rising gas prices [13].

Thus, reducing temperature in buildings could save gas *and* money for consumers, emphasizing the political feasibility and potential win-win benefits of the measure. Even better, it could be implemented relatively easily and quickly compared to investment measures such as increasing wind and solar power or major building refurbishment. This would enable rapid contributions to managing the current gas crisis.

### 1.2 State of knowledge

Ruhnau et al. empirically estimated the response of natural gas consumers in Germany regarding the energy crisis at the national level [14]. They found that households and small businesses experience significant reductions in gas consumption from March 2022 onwards, with relative savings of up to 28 % below the temperature-adjusted baseline. They show that the observed changes in natural gas consumption correlate with rising natural gas prices. While the authors did not explicitly identify the drivers of the gas savings, they do provide some indication of the influence of room temperature behavior, suggesting the potential for thermostat adjustments.

Initial evaluations of adjusting thermostats also indicate a promising effect in terms of gas savings at the energy system level [7,9,10] (see also Fig. 1). However, these assessments are quite undifferentiated and only state a snapshot saving potential at the system level. In particular, these estimations do not take into consideration the different drivers of gas consumption and corresponding saving potentials in buildings.

For instance, gas consumption is highly dependent on building characteristics such as size, age and residence type [[15], [16], [17]]. In addition, it was shown that buildings’ thermal response to reducing temperature setpoints is strongly influenced by building age and refurbishment status [18]. Finally, weather can be a decisive factor for residential gas demand [19,20]. It follows that the gas savings potential is also dependent on building characteristics, temperature setpoints as well as weather.

### 1.3 Approach and contribution

This paper aims to go beyond such undifferentiated snapshots at the system level. Hence, we calculated gas consumption as well as corresponding gas and CO_2_ saving potentials for different building types, temperature setpoints and weather years. To this end, we applied a set of validated reduced-order models of the thermal behavior of building archetypes that we developed in a previous study [18]. Furthermore, we contrasted the estimated gas saving potential with the gas costs and monetary savings of individual consumers, considering different price scenarios for natural gas.

Our approach tries to answer three pressing questions of high political relevance. First, at a national level, *how much natural gas can be saved annually by lowering heating temperature setpoints in buildings, considering building characteristics and weather dependencies*? With these more nuanced calculations, initial estimations are substantiated by considering different drivers of saving potentials, and the “low hanging fruits” for saving natural gas in the short term are revealed. Once it is understood how different building types "react" to temperature reduction in terms of gas consumption, targeted policy measures can be derived.

Second, *how are CO*_*2*_ *emissions affected by lowering temperature setpoints*? This allow us to assess whether, beyond their short-term contribution to the gas crisis, thermostat adjustments are an important medium-term measure for climate protection.

Third, on an individual consumer level, *how large is the influence of adjusting thermostats with regard to the consumer's energy bill*? Can “freezing” offset high prices of natural gas use that may persist even after the end of the war due to CO_2_ pricing instruments?

Exploring the consumer's economic and financial perspective is important for assessing how likely the saving potential will be realized, since adjusting thermostats in residential buildings is a voluntary measure that cannot be politically enforced. Furthermore, it is crucial to measure the monetary impact on consumers, both to gauge the acceptability of the saving measure and to determine the need for consumer relief due to surging prices. Finally, this will provide an indication of the extent to which certain groups of households may be at risk of energy poverty.

Our calculations focus on Germany. It is the largest consumer of natural gas in the European Union with an annual consumption of around 870 TWh in 2020 [21]. More than half of the 40 million apartments in Germany are heated with natural gas [22]. Thus, many households are affected by the gas crisis and the corresponding surge in prices. Nevertheless, the trends of our results as well as their implications are transferable to other countries, especially those located in similar climatic zones and comparable building structures.

The remainder of this paper is structured as follows. Chapter 2 describes the methods we used to determine gas consumption and associated savings potentials, CO_2_ emissions and consumer cost. Chapter 3 presents and discusses the results on gas consumption at the building and system level (section 3.1), on the induced CO_2_ savings (section 3.2) and on the costs for consumers (section 3.3), followed by a discussion of implications for policymakers (section 3.4) and of the limitations of our approach (section 3.5). Finally, conclusions are drawn in chapter 4.

## 2 Methods

This chapter first describes the computation of heating demands of typical buildings depending on temperature setpoints (section 2.1). Section 2.2 then explains how these heating demands are translated into gas consumption for individual buildings as well as into potential savings at the national level. The computation of induced CO_2_ savings is described in section 2.3, and finally, the calculation of consumer cost is addressed in section 2.4.

### 2.1 Heat demand calculation for typical buildings

To adequately determine saving potentials as a function of building characteristics, temperature setpoints and weather, we used a set of validated reduced-order bottom-up models of building thermodynamics [18]. These models compute the space heating demand of buildings for given weather data and temperature setpoints in a high temporal resolution (e.g. 15 min). Building thermodynamics are described by lumped resistance-capacitance thermal networks, accounting for transmission and ventilation losses, solar and internal heat gains as well as thermal inertia. Models are available for twelve typical German single-family houses (SFH) in three insulation states that differ in building age, size, construction materials and heating distribution systems. Thus, they represent the stock of German SFH. Compared to thermal simulation software like, e.g., TRNSYS [23], the reduced-order models are computationally highly efficient and allow multiple parameter variations. The development and validation as well as the parameters of the models is described in detail in Ref. [18]. A summarized overview of the resistance-capacitance thermal network models employed in this study is available in the Appendix.

The buildings considered are taken from the German residential building typology [24]. The focus is on detached SFH, because these are the most prevalent building category with approx. 10 million detached SFH out of 19 million buildings in total [24]. In addition to that, detached houses generally have higher specific gas consumption levels than other types of dwellings [15]. Table 1 provides an overview of the building types as well as their stock in Germany. In the remainder of our study, the labelling of the building types is after their construction period. For each of these basic types, three variants are specified, which differ according to their energy refurbishment level: 1) the status quo condition, i.e. not or only marginally refurbished, 2) the condition after moderate energy refurbishment in accordance with legal minimum standards, and 3) the condition after ambitious energy refurbishment that corresponds to the usual insulation standards for passive houses.

**Table 1:** Stock of SFH according to the German residential building typology [24].

Code of building type acc. to original study	Building type label in our study	Construction period	Heated living area in m^2^	Building stock in thousands
SFH A	<1859	<1859	199	330
SFH B	<1919	1860–1918	129	966
SFH C	<1949	1919–1948	275	1131
SFH D	<1958	1949–1957	101	859
SFH E	<1969	1958–1968	110	1509
SFH F	<1979	1969–1978	158	1507
SFH G	<1984	1979–1983	196	704
SFH H	<1995	1984–1994	137	1160
SFH I	<2002	1995–2001	111	1035
SFH J	<2010	2002–2009	133	907^a^
SFH K	<2016	2010–2015	160	494^a^
SFH L	<2022	>2016	160	507^b^

a numbers derived from Federal Statistical Office [25].

b stock end of 2021.

To investigate the effect of temperature reductions, we varied the indoor air temperature setpoints for the heating period between 17 °C and 23 °C in 1 °C steps. This setpoint is assumed to be applicable at daytime between 6 a.m. and 10 p.m. For the remaining time, we assumed a night setback temperature that is 0.85 times the day temperature setpoint [24]. For the sake of simplification, we considered an average temperature for the whole building, i.e. we did not account for different thermal zones. Internal gains are assumed to remain constant at 3 W/m^2^ [24].

The reduced-order models require ambient temperature and total solar radiation on the southern vertical surface as input data. While the ambient temperature is important for transmission and ventilation losses, the solar radiation determines solar gains within the building. We retrieved ambient temperature, surface solar radiation downwards as well as total sky direct solar radiation at surface from ERA5 reanalysis data [26] for the city of Frankfurt am Main, Germany. Solar radiation data was then processed with TRNSYS to the required orientation. We employed two different weather data sets – 2010 and 2018 – to determine the range of energy demand between particularly cold and particularly warm years. The year 2010 is a typical representative of a cold year in the recent past and 2018 is a warm one according to the heating degree days for the selected climate^1^ [27]. Furthermore, there are differences in solar radiation between the two years, resulting in higher solar gains for the warmer year.^2^

### 2.2 Calculation of gas consumption

The calculated annual building-level heat demands were transformed to natural gas demands by dividing by building type specific gas heater efficiencies. They differ according to the building construction period and refurbishment levels, since these are decisive factors for the heating system temperatures [18] and hence for efficiencies. The parameters used in the calculations are given in Table 2.

**Table 2:** Gas boiler parameters [24,28].

Building types	Refurbishment level^a^	Heater technology	Efficiency
**<1859 – <2002**	SQ	Low-temperature boiler	83 %
**<1859 – <2002**	MR, AR	Condensing boiler	91 %
**<2016 – <2022**	SQ, MR, AR	Condensing boiler	93 %

^a^ Abbreviations: SQ = status quo, MR = moderately refurbished, AR = ambitiously refurbished.

These gas *demands* were then multiplied with a building-specific correction factor to adjust them to typical gas *consumption* values, in accordance with the procedure that also underlies the German residential building typology [24]. This correction factor varies between 1.1 and 0.6 depending on the area-specific gas demand and is lower for buildings with high demands. It addresses the fact that the measured energy consumption is on average accordingly lower than the calculated demand, especially for poor energy standards [24] – possibly due to different user behavior depending on the energetic building condition [29].

Finally, building-level gas consumptions were extrapolated to the nation-wide stock of SFH with statistical data on the number of buildings (Table 1) as well as the corresponding gas heater market penetration (Table 3).

**Table 3:** Gas heater market penetration by building age [30].

Building types	Gas heater market penetration
**<1859 – <1949**	63 %
**<1958 – <1979**	47 %
**<1984 – <1995**	49 %
**<2002 – <2010**	65 %
**<2016 – <2022**	46 %

### 2.3 Estimation of induced CO_2_ savings

CO_2_ emissions as well as savings due to thermostat adjustments at system level were calculated by multiplying the aggregated gas consumption and related savings per temperature setpoint with the CO_2_ emission factor for natural gas from Russia. The latter is derived from Juhrich, 2016 [31] and amounts to 198.6 tCO_2_/GWh.

### 2.4 Computation of consumer cost

Gas cost on building level were computed for different gas price scenario assumptions (Table 4). While the scenario “LOW” reflects the average price for natural gas for German households at pre-crisis level in the second half of 2021 [32] and which is still valid for many longer-term contracts, the medium scenario “MED” corresponds to the average price level for households in April 2023 [6]. As an upper level, the scenario “HIGH” represents average prices for new customers during a high-price period in August 2022 [33]. Fixed price components that apply irrespective of gas consumption level or price tariffs that are dependent on the consumption level were not considered in this analysis.

**Table 4:** Natural gas retail prices for different scenarios considered [6,32,33].

Scenario	Price level
LOW	6.9 €ct/kWh
MED	14.0 €ct/kWh
HIGH	40.0 €ct/kWh

## 3 Results and discussion

We describe and discuss our results in five parts. While the first part deals with gas consumption levels and corresponding saving potentials at the building and the system level (section 3.1), the second part shows induced CO_2_ savings (section 3.2). Section 3.3 deals with the consumer's economic and financial perspective. We discuss the implications for policymakers in section 3.4, and finally state our limitations in section 3.5.

### 3.1 Gas consumption and saving potential

**Fig. 2:**
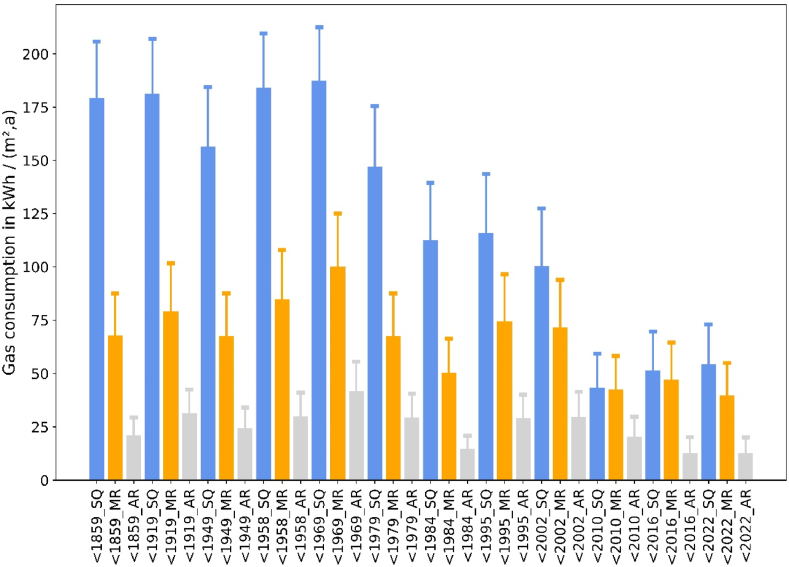
Specific gas consumption for space heating by building type for a temperature setpoint of 20 °C (base weather year = 2018, error bars = 2010. Abbreviations: SQ = status quo, MR = moderately refurbished, AR = ambitiously refurbished).

We first consider gas consumption at building level in order to highlight the dependence of gas consumption for space heating on building characteristics such as their construction period and insulation levels. Therefore, we present the annual gas consumption for space heating for different building types for a fixed setpoint of 20 °C (Fig. 2). Consumption is shown for the warmer year 2018, while error bars indicate consumption levels for the cold year 2010. Consistent with a number of studies on residential gas consumption [[15], [16], [17],^19^], our results show that consumption is higher in old buildings (left in the diagram). Gas consumption decreases with the insulation level, i.e. status quo buildings (blue bars) consume most, followed by moderately refurbished buildings (orange) and ambitiously refurbished buildings (grey). The annual gas consumption varies between 12 and 187 kWh/(m^2^,a) for 2018.

In line with literature [19,20], our results indicate that weather has a large influence on gas consumption. Across all building types, consumption in the cold year 2010 is about 32 % higher on average.

For the analysis of the possible gas savings by adjusting thermostats, we now focus on the status quo energetic condition (“SQ”) for the buildings, since this is decisive for short-term changes in gas consumption.

**Fig. 3:**
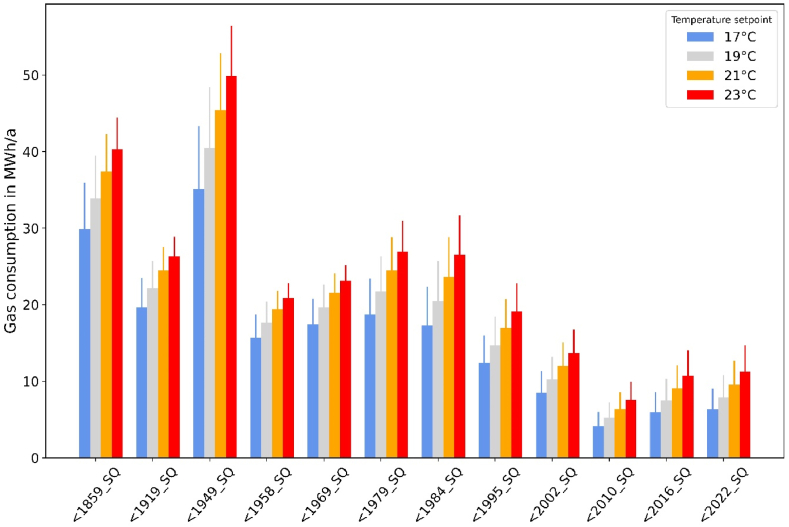
Gas consumption for space heating by building type and temperature setpoint (base weather year = 2018, error bars = 2010).

Fig. 3 shows gas consumption for space heating of different building types as a function of the temperature setpoint.

Compared to heating at 17 °C, the gas consumption for heating at 23 °C is around one third higher for old buildings constructed before 1969 and almost twice as high for newer buildings constructed after 2002. For the high-consuming building type “<1949_SQ”, for instance, this corresponds to a difference of 15 MWh/a (weather year 2018). In principle, the *absolute* savings potentials (in MWh/a) by turning down thermostats are significantly greater for older buildings, while *relative* savings (in percent) tend to be higher in the newer building types. The latter is possibly due to the fact that newer, well insulated buildings profit more from free heating sources like internal or solar gains, which cover the remaining heating demand at reduced setpoints disproportionately high.

**Fig. 4:**
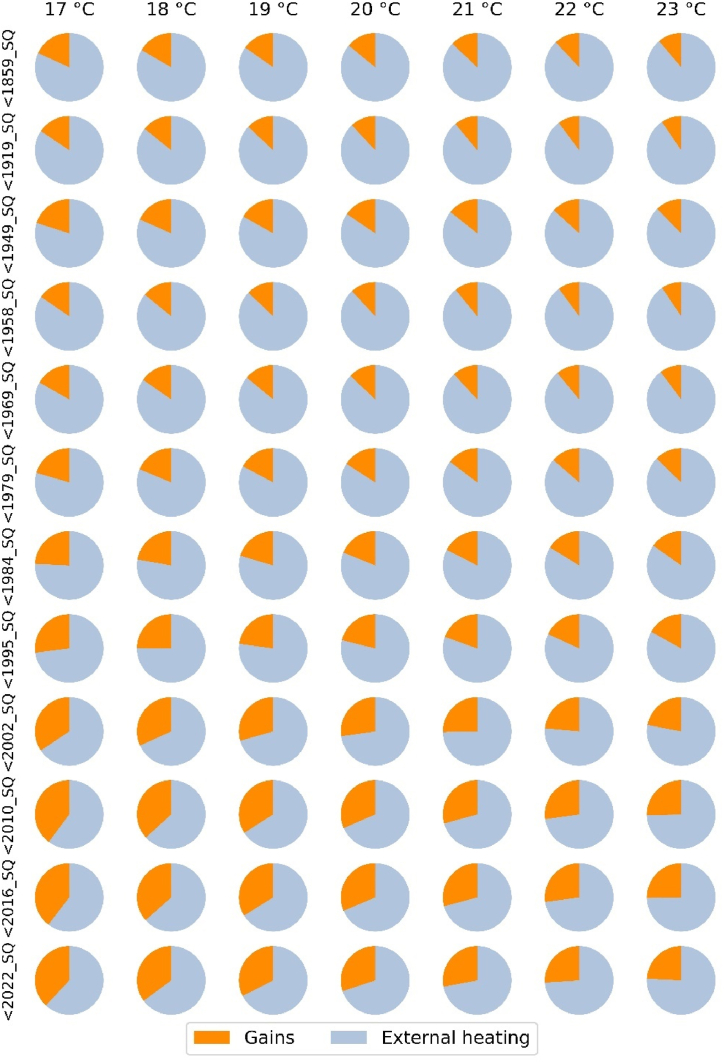
Breakdown of total heat supply by (internal and solar) gains and external heating (e.g. by natural gas) for building types and setpoints (weather year 2018).

This is illustrated in Fig. 4. It shows, for each building type and setpoint, how internal and solar gains contribute to the total heat supply. The proportion of free heating sources increases with decreasing building age and decreasing initial indoor temperature. In newer buildings, internal and solar gains can account for up to 25 %–40 % depending on the setpoint, while in older buildings they account for only about 10 %–15 %.

**Fig. 5:**
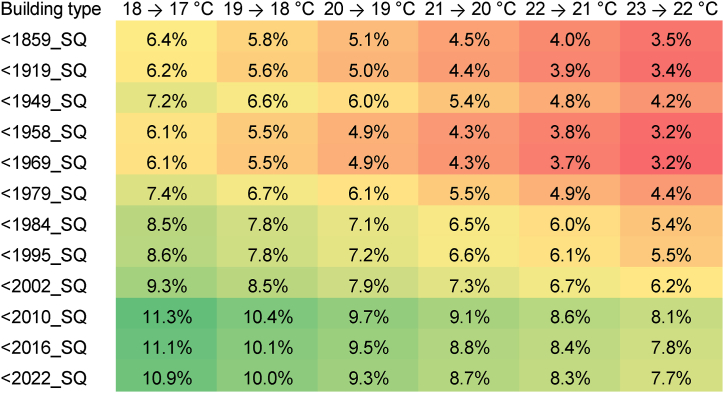
Annual gas savings per °C temperature setpoint reduction (weather year 2018).

Note that the lower the initial temperature setpoint is before reduction, the higher the relative saving potential. This is due to reduced transmission and ventilation losses at lower indoor temperatures. Fig. 5 illustrates this relationship by indicating relative savings from lowering the heating temperature setpoint by 1 °C compared to different initial temperatures. The relative saving potential ranges from 3 % annually for older buildings with high initial temperatures to more than 11 % annually for newer buildings with low initial temperatures. By lowering the room temperature from 21 to 20 °C, 6.3 % of gas can be saved per year on average across all building types.

**Fig. 6:**
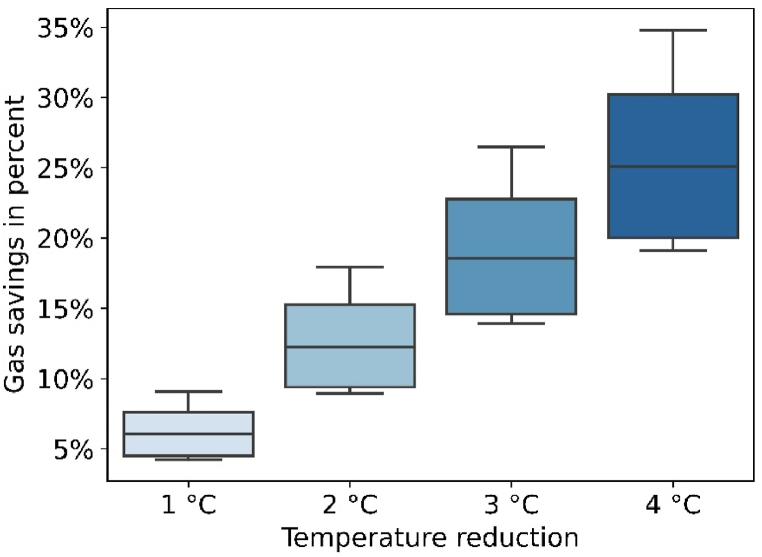
Annual gas savings for variable temperature reduction levels across all building types (initial temperature: 21 °C, weather year 2018).

Savings effects due to higher temperature reduction levels are shown in Fig. 6. The range of savings across all building types is shown, provided that the temperature is reduced from 21 °C to lower temperatures. On average, they account for 12 % for 2 °C reduction to 26 % for 4 °C reduction. Some buildings (particularly newer ones) can save 18 % of gas by lowering temperatures by 2 °C. Other (older) buildings only achieve this level of savings with a reduction of 4 °C. The savings increase largely linearly with the amount of temperature reduction.

**Fig. 7:**
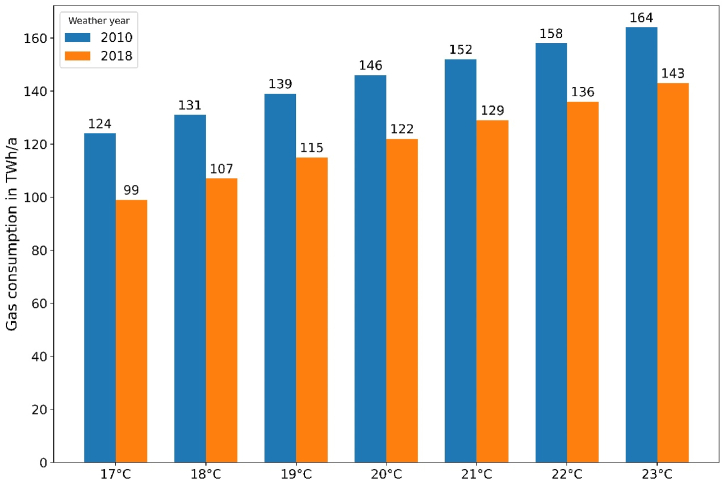
Aggregated gas consumption for stock of German SFH by temperature setpoint and weather year.

Of particular relevance to energy policy are the extrapolated gas consumption and savings potentials for the entire stock of SFH, because these determine the overall effectiveness of the measure. Therefore, Fig. 7 shows the *aggregated* gas consumption for space heating for different temperature setpoints and weather years. For a setpoint of 21 °C, the total gas consumption for space heating amounts to between 129 TWh/a (weather year 2018) and 152 TWh/a (weather year 2010).

While using validated *models* for the calculation of gas demands, a validation of this *result* was not possible due to the lack of statistical data concerning gas consumption in different building types. However, a plausibility check can be made with the assumption that SFH have twice as high a space heating demand as apartment buildings [34]. Based on our results for the year 2018 (Fig. 7) and assuming an average room temperature of 21 °C,^3^ the total gas consumption for domestic space heating (SFH plus apartment buildings) would amount to 194 TWh/a. According to the energy statistics [21], consumption was 202 TWh/a in 2018 – an acceptable deviation of only 4 %.

**Fig. 8:**
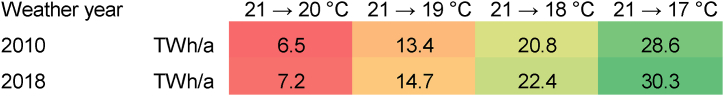
Total gas savings for variable temperature reduction levels.

The potential savings of natural gas when turning down thermostats from 21 °C to lower temperatures are shown in Fig. 8: for the entire stock of German SFH, about 14 TWh/a (equivalent to around 10 %) of natural gas can be saved with a temperature reduction to 19 °C. With a massive reduction to 17 °C, the savings could even amount to approximately 30 TWh/a (around 20 %).

Based on the Russian natural gas import volume of 495 TWh/a before the crisis [10], up to 6 % could be saved by a large reduction of temperature setpoints in SFH from 21 to 17 °C. A more moderate setpoint reduction to 19 °C could bridge the import gap to nearly 3 %.

Somewhat counterintuitively, absolute gas savings by reducing temperatures are slightly higher for warmer weather. This can be explained by lower losses through the building envelope at higher ambient temperatures; the remaining heating demand can then be covered to a larger extent by heat gains inside the buildings.^4^ Generally, the absolute savings are quite independent of the weather year.

Our estimations regarding the gas saving potential are generally lower than findings of other studies for comparable temperature reduction levels (Table 5). This is possibly due to the fact that we consider space heating in SFH only, while the other studies estimate saving potentials for the total or at least a larger fraction of the building stock. To get a comparable basis, consider that SFH account for about half of the total energy demand for space heating in Germany [35]. As an approximation – data on gas heater penetration is not available for German commercial buildings – the gas saving potential for the entire building stock in Germany can thus be estimated to be twice as high as for SFH only, i.e. approximately 14–28 TWh/a for a setpoint reduction of 1–2 °C. With these considerations in mind, our results on the saving potential at system level are in line with the studies mentioned in Table 5. However, our study goes far beyond the mentioned studies by demonstrating important dependencies of gas savings on e.g. building types and weather, and by contrasting the individual consumer's perspective.

**Table 5:** Results of various studies on gas savings effects through temperature reduction in buildings.

Study	Quantification method for gas savings	Buildings considered	Temperature reduction level	Gas saving potential in TWh/a
Agora Energiewende, 2022 [9]	Top down by lumped saving factor	All buildings	0.5–1.0 °C 1.0–1.5 °C	14 22
Forschungszentrum Jülich, 2022 [10]	Top down by ratio of temperature setpoints	Residential buildings	1.0–2.0 °C	40^a^
Our study	Bottom up considering building characteristics	Single-family houses	1.0 °C 2.0 °C	7 14

a includes effect by substituting gas boilers for domestic hot water generation by electrically powered instantaneous water heaters.

### 3.2 Induced CO_2_ savings

**Fig. 9:**
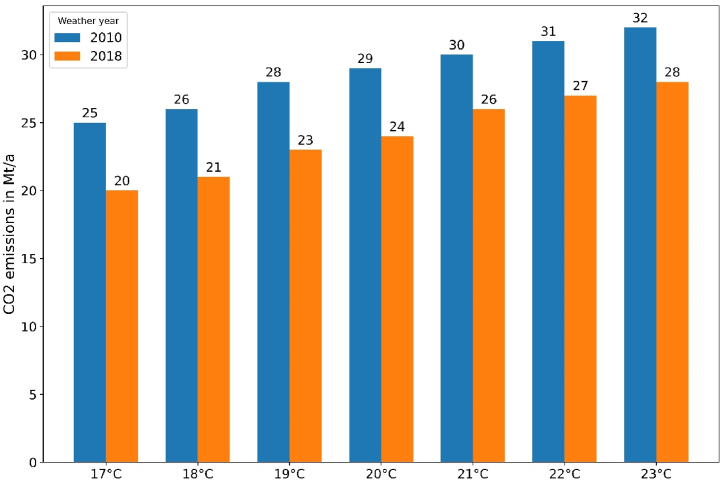
Annual CO_2_ emissions for stock of German SFH by temperature setpoint and weather year.

The temperature setpoint for space heating not only affects gas consumption, but also CO_2_ emissions. Fig. 9 shows the aggregated CO_2_ emissions for the stock of German SFH for different setpoints and weather years. They are proportional to the gas consumption at system level and sum up to 26 Mt/a (weather year 2018) or 30 Mt/a (weather year 2010) for a setpoint of 21 °C.

**Fig. 10:**
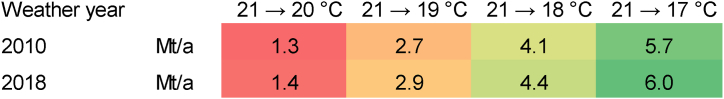
Total CO_2_ savings for variable temperature reduction levels.

Fig. 10 reveals the total CO_2_ saving potential at system level for increasing temperature reduction levels for an initial temperature of 21 °C. Based on the annual CO_2_ emission level of German households of 90 Mt/a [21], 3 % and up to 6 % could be saved by a setpoint reduction of 2 °C–4 °C. This means that thermostat adjustments are an important flanking measure for climate protection beyond the current gas crisis.

### 3.3 Economic and financial considerations

**Fig. 11:**
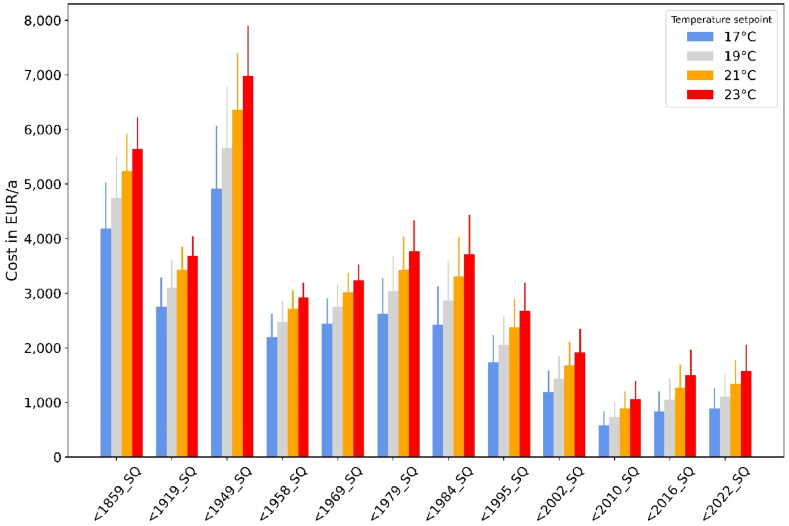
Gas bill for space heating by building type and temperature setpoint for the medium gas price (14 €ct/kWh; base weather year = 2018, error bars = 2010).

From an individual consumer's perspective, gas cost and corresponding saving potentials are directly proportional to consumption (see Fig. 11). Assuming a medium gas price of 14 €ct/kWh, the annual bill amounts to 4900 € to 6980 € depending on the temperature setpoint for old and large buildings (building type “<1949_SQ”, weather year 2018). For smaller and well insulated buildings (“< 2010_SQ”), only 580 to 1060 €/a apply. For a reduction of temperature setpoints from 21 to 20 °C, the annual bill can be reduced by 4–9 % depending on the building type. This translates to 340 €/a of annual savings for building type “<1949_SQ” and to 80 to 120 €/a for newer buildings.

**Fig. 12:**
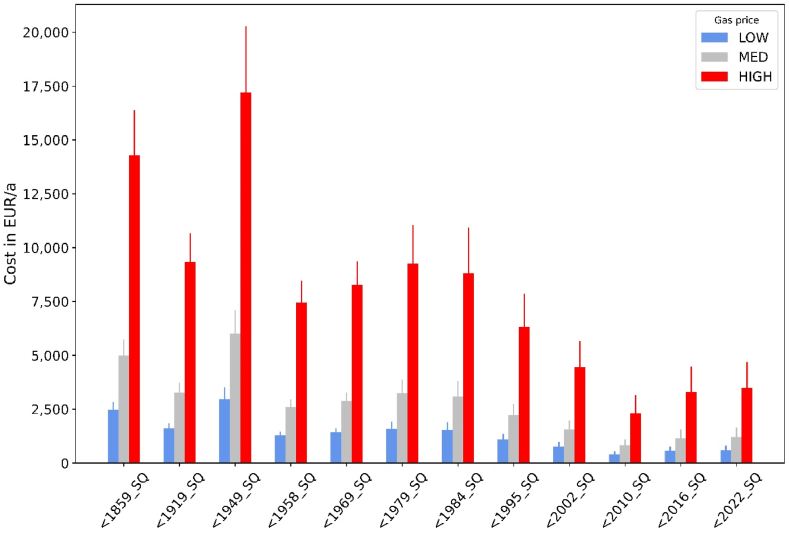
Gas bill for space heating by building type and gas price at a fixed temperature of 20 °C (base weather year = 2018, error bars = 2010).

However, as Fig. 12 demonstrates, consumer costs are highly sensitive to gas prices. Annual bills can reach up to 20,000 €/a for old and large buildings in cold winters if gas prices surge to 40 €ct/kWh. Even residents of energetically better buildings in such cases have to spend more than 3000 €/a on space heating. If the average consumption expenditure of German households is taken as a basis [36], annual spending for space heating could rise from 2 % (before the gas crisis) to 10 % for new buildings and from 12 % to even 67 % for large and old buildings in this scenario. Compared to pre-crisis levels (scenario “LOW”), annual bills would then be almost six times higher. Note that these results consider only gas consumption for space heating. Of course, the total consumer bill could be even higher if natural gas is also consumed for domestic hot water generation.

The scale and impact of this financial burden becomes particularly clear when it is linked to the income structure by building type. The lower the income, the more likely households are to live in an older building [37,38]. Low-income households are already severely affected by energy costs. Rising energy prices due to the energy crisis exacerbate the situation and push these households to the brink of energy poverty.

**Fig. 13:**
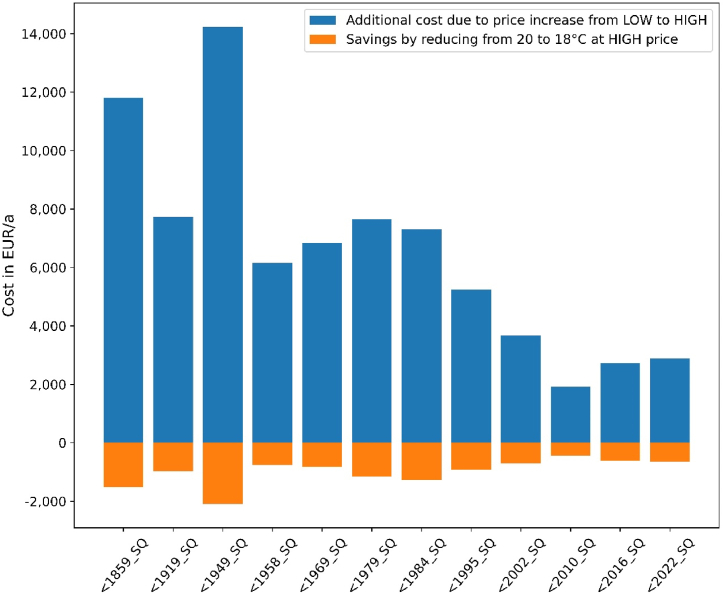
Additional gas cost due to price increases from LOW to HIGH for a fixed temperature of 20 °C versus savings by turning down thermostats from 20 to 18 °C (weather year = 2018).

Thermostat adjustments can unfortunately lower the consumer bill only to a small extent. As shown in Fig. 13, the additional cost for consumers due to an increase of prices from pre-crisis levels (“LOW”) to high prices (“HIGH”) outweigh potential savings by turning down thermostats from 20 °C to 18 °C by far. Inhabitants of newer buildings are better off: while price-induced cost increases amount to 480 % for all building types, cost savings by reducing temperature by 2 °C at high prices amount up to 20 % in newer buildings but only to 10 % in older ones.

This again shows that low-income households are particularly affected, as they tend to live in older buildings [37,38]. Whether these particularly vulnerable households benefit at all from thermostat adjustments – as they may already be heating their homes to low temperatures – cannot be conclusively established by the present analysis. This is due to the lack of combined data on income, building age, type and size, heating behavior and level of refurbishment.

### 3.4 Implications for policymakers

Our results have two major implications for policymakers. First, thermostat adjustments are an important no-regret measure for reducing the gas dependency at the system level. Our findings support initial estimations on the effectiveness of adjusting thermostats in terms of gas savings. On top of that, we could show that while gas *consumption* is highly weather dependent, the absolute *saving* potentials at system level do not vary significantly for different weather conditions and are thus quite robust.

Compared to other measures like building refurbishment or renewable energy expansion, thermostat adjustments come at no investment cost and can be realized immediately without any time lags due to, e.g. delivery bottlenecks or shortage of skilled workers, which might apply for other gas saving measures. Thus, thermostat adjustment could also help to bridge possible gas shortages in the following winters at short notice, while more structural measures such as heat pump installations will only be available to a limited extent. In addition, thermostat adjustments have important side-benefits with regard to their CO_2_ saving potential. Finally, as gas prices are also expected to increase in many countries due to CO_2_ pricing mechanisms, thermostat adjustments will remain an important financial saving measure for many households.

For the planning and assessment of corresponding political instruments, our building-specific results should be considered. On the one hand, we could show that the lion's share of possible absolute gas savings is with old and large buildings, especially those constructed between 1919 and 1948 (which are, in addition to that, very prevalent in Germany). At the same time, these building types suffer the most economically by rising gas prices. Thus, there is probably a high incentive for voluntary demand reduction. On the other hand, monetary benefits of gas savings are limited for newer and energetically better buildings, thus also threatening the acceptability of the measure for these customers. As a consequence, the realizable gas saving potential could have to be corrected downwards by the (small) contribution of these newer buildings.

Furthermore, policymakers should take into consideration that gas savings due to temperature reduction in residential buildings cannot be subject to obligation, since each homeowner controls his or her own heating. Therefore, policymakers should raise awareness among households on the saving effects of thermostat adjustments – not only to ensure energy security, but also to save money and to contribute to climate protection.

Finally, many consumers are not yet exposed to scarcity prices due to long-term contracts with their suppliers. Thus, we think it is important that policymakers and energy suppliers provide early information about possible price increases, as a high gas price can be considered as an important signal to reduce consumption [39].

Our second major policy implication addresses the financial burden of rising gas prices for consumers, which is significantly higher for older buildings than for younger ones. We therefore conclude that households should be relieved financially according to building type. Our results provide suggestions for the design, possible impacts as well as governmental expenditures of such consumer relief measures.

A relief model that is currently discussed for many European countries is the introduction of a discounted basic energy consumption for households. For instance, Germany provides customers a state-guaranteed price for 80 % of the previous year's gas consumption. For the remaining 20 % of gas consumption, the (probably high) market price must be paid [40]. Austria, in contrast, has implemented an electricity cost subsidy based on a fixed basic consumption, i.e. only consumption above a fixed threshold must be paid for at market prices [41]. Similar models for space heating with natural gas could follow.

If such models were implemented, our findings imply that, depending on the building type, there could be a high level of inequality for the residual consumption that is exposed to high market prices. Taking the German proposal as an example, newer buildings could realistically avoid the market price by reducing their temperature by slightly more than 2 °C, as this corresponds to a 20 % saving. However, for older buildings, which are substantially more affected by the crisis, a 20 % saving is very ambitious. It translates into a 4 °C reduction, which may have side effects such as negative health. Models that rely on a fixed basic consumption, like the Austrian measure for electricity consumption, are to be regarded as particularly vulnerable in terms of distributive justice in view of our findings. Energetically poor buildings, for example, could hardly benefit from a subsidized basic consumption (at least if this is oriented at average consumption levels), while newer buildings could be overcompensated and make high windfall profits.

Based on our findings, it could be worth considering the justification for the government subsidy on gas usage depending on building type in order to achieve a more balanced policy. Consequently, a standardized reduction in temperature could potentially be established as a fair benchmark for the various building types. For example, targeting a reduction of 2 °C across all building types would result in a decrease in gas consumption by approximately 10 % for older buildings and around 20 % for new buildings (refer to section 3.1). Therefore, unrenovated older buildings should receive a guarantee from the government for up to 90 % of their gas consumption. In contrast, newer and more energy-efficient buildings should only receive subsidies for 80 % of their consumption.

This is all the more true given that old, unrenovated buildings are more likely to be occupied by low-income households [37]. This means that aspects of distributive justice have to be considered, for several reasons. First, after energy-related retrofitting buildings, a study could show that despite energy consumption was reduced by 70 %, a third of residents still faced higher costs resulting in an unforeseen financial burden [42]. Second, retrofitting may even lead to displacement of low-income residents, contributing to conflicts and injustice [43]. Third, although less economically privileged groups have the lowest absolute levels of energy consumption, they show the highest rebound effects [44]. Policies should focus on the former, not the latter.

However, there are already policy instruments that operationalize Rawl's ideas of distributive justice for the energy sector, measuring the distribution of energy consumption [45]. This suggests that in order to design socially and distributionally just compensation policies, our results would ideally need to be combined with other, granular data to identify the most vulnerable households that need higher compensation. These include income [46], health [47] and age of occupants [38].

By integrating the technical and economic dimension with the social dimension, sustainable solutions can be developed to prevent energy poverty – even beyond the short-term contribution to the current gas crisis [38,48]. However, the implementation of this integrative analysis is beyond the scope of our study.

### 3.5 Limitations

The results and their implications should be interpreted in the light of the limitations of this study.

First, we demonstrated a novel bottom-up method for the calculation of gas savings, taking the example of German detached SFH only. While we consider the national focus very relevant given Germany's high importance in terms of overall gas consumption and import dependency on Russia (compare chapter 1), the focus on the building category was because of its relevance in energetic terms (compare chapter 2.1). Nevertheless, we believe that our main findings are transferable to other countries and building categories.

This is especially true for our general finding that gas consumption as well as induced gas savings, CO_2_ emissions and cost are strongly dependent on the building type, and hence, that political measures should be planned accordingly. We consider these findings particularly transferable for countries located in similar climate zones and that are dependent on natural gas for space heating, such as the Netherlands, United Kingdom, Slovakia or Italy [12]. Finally, as our method is easily adaptable to other climates, further studies could analyze gas savings by thermostat adjustments in different countries with similar building conditions.

Second, when computing the total gas savings potential, we considered only buildings in the status quo energetic condition according to the German residential building typology. This means that buildings that have been refurbished were not taken into account since there is little granular data available on the renovation status of typical German residential buildings. In contrast, results are available at the aggregate level of the German building stock: about 19 % of the exterior wall surface and as much as 37 % of the roof surface of German residential buildings have been retrofitted with insulation [49]. Given these figures, our results may be overestimated. However, due to the described demand-consumption adjustment, the potentially overestimated standard consumption of old, uninsulated buildings should be relativized. Furthermore, our results at the national level are quite consistent with national statistical data (see section 3.1).

Finally, our study was limited to the evaluation of the effects of temperature reduction in buildings regarding gas consumption, CO_2_ emissions and cost. This means that possible side effects of adjusting thermostats were not considered, in particular health effects [50], thermal comfort violation [51] and mold growth [52].

While we believe these limitations have not biased the primary outcome of our work, future studies could seek to include better data on the buildings’ level of refurbishment. These could improve the determination of potential gas savings and financial burden. Avenues for future research include the integration of behavioral science in terms of thermal comfort, heating habits and the acceptability of thermostat adjustments, or considering the related price-elasticity. Regarding the impact on consumer bills, it could be interesting to include socio-economic drivers of residential gas consumption such as income in order to address the distributive justice of possible relief measures. Such granular data could also help design socially equitable compensation policies and prevent energy poverty among the most vulnerable groups.

## 4 Conclusion

Facing an international energy crisis, various policy measures are currently discussed in order to reduce imports of natural gas and to provide financial relief for private households due to higher gas prices. This paper provides an in-depth analysis of thermostat adjustments in residential buildings, which might be one of the most effective measures for saving gas in the short term. In order to shed light on this measure from different perspectives, we used a novel bottom-up approach to calculate gas consumption as well as induced gas, CO_2_ and cost savings subject to varying temperature setpoints and weather conditions at building archetype level. We demonstrated this approach using the example of German single-family houses.

Our study confirms a promising potential for natural gas savings by thermostat adjustment in German single-family houses. According to our calculations, about 14–30 TWh/a of natural gas could be saved if temperature setpoints are reduced by 2 or 4 °C. This corresponds to 3–6 % of Russian gas imports to Germany as of 2020.

Our results indicate significant differences in gas savings depending on building characteristics such as age and size. *Relative* savings vary between 4 and 9 % if thermostats are turned down from 21 °C to 20 °C, and are higher for newer buildings. However, the biggest *absolute* savings are possible for old and large buildings. Furthermore, savings are found to be highly dependent on temperature settings, i.e. they are greater for lower initial temperatures prior to setpoint reduction.

From an emission perspective, about 3–6 Mt/a of CO_2_ savings are induced by turning down thermostats by 2–4 °C. This is equivalent to 3–6 % of German household's annual CO_2_ emissions. As such, setpoint reduction can be an import measure also beyond the upcoming winter(s).

Taking an individual consumer's perspective, we calculated that 80 to 340 €/a can be saved with a thermostat reduction by 1 °C at a gas price of 14 €ct/kWh. The older and larger the building, the higher the absolute savings. The increased gas prices are a considerable burden on private households. However, the results of our investigations show that households are affected differently depending on the type of building: space heating could account for 10 % for new buildings and even for 67 % for large and old buildings of average consumer spending in Germany in a worst-case scenario (high natural gas price, cold winter). Generally, monetary savings due to thermostat adjustments do not outweigh surge prices in the course of the crisis.

Our results suggest that thermostat adjustments should be promoted by policymakers – not only in Germany, but in countries around the world with similar climatic conditions and building structures. They are an effective and relatively simple measure to implement to make Germany and Europe more independent of Russian natural gas in the short term and – at the same time – to relieve consumers. In addition, they serve as a climate protection measure.

On the other hand, the results show that it is crucial to provide further relief for consumers if natural gas prices rise sharply. Building characteristics should be considered since old and large buildings are particularly affected by the crisis-related energy price shocks. At the same time, social aspects such as the income of the affected households should be included to achieve distributive justice and avoid energy poverty.

Thermostat adjustments could be an important measure to reduce gas consumption for many countries in Europe. However, given the overall gas supply gap, thermostat adjustments alone cannot be the only way out of the gas crisis. Other measures are needed to reduce the import dependencies, such as the energy-efficient renovation of buildings or the expansion of wind and solar. In existing buildings, gas-based heating systems could eventually be supported by heat pumps or solar thermal, combining the benefits of fossil fuels and renewables. The question is whether these more structural measures can be implemented quickly enough to close the gap.

## Data availability statement

Data associated with this study has been deposited on Zenodo at 10.5281/zenodo.10008150.

## CRediT authorship contribution statement

**Evelyn Sperber:** Writing - original draft, Visualization, Validation, Software, Methodology, Investigation, Formal analysis, Data curation, Conceptualization. **Ulrich Frey:** Writing - review & editing, Supervision, Conceptualization. **Valentin Bertsch:** Writing - review & editing, Supervision, Conceptualization.

## Declaration of competing interest

The authors declare that they have no known competing financial interests or personal relationships that could have appeared to influence the work reported in this paper.

## Figures and Tables

**Fig. 14:**
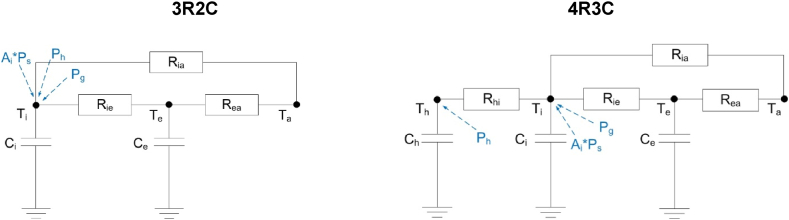
Thermal network representations for building types. Abbreviations: T_i_ = indoor air temperature, T_e_ = temperature of building envelope, T_h_ = temperature of heaters, T_a_ = ambient temperature, P_h_ = heating power, P_g_ = internal heat gains, P_s_ = solar radiation, R_ia_ = thermal resistance between interior and the ambient, R_ie_ = thermal resistance between interior and building envelope, R_ea_ = thermal resistance between building envelope and the ambient, R_hi_ = thermal resistance between heaters and interior, C_i_ = capacitance of interior, C_e_ = capacitance of building envelope, C_h_ = capacitance of heaters.

## Appendix

### Appendix A Resistance-capacitance thermal network models

In this study, two distinct resistance-capacitance thermal network models are utilized to calculate heat demand based on the building type. These models, retrieved from Ref. [18], are different for buildings erected before 2002, which are defined by the 3R2C model (Fig. 14, left), and for newer building types which are accounted for by the 4R3C model (Fig. 14, right). The underfloor heating system in the latter buildings leads to higher thermal inertia. C represents the effective thermal masses of the model nodes, whilst R represents the thermal resistances between them.

## Footnotes

1 Heating degree days amount to 2371 in 2010 and to 1768 in 2018 [27].

2 Specifically, the annual amount of surface solar radiation downwards at the given site was 1127 kWh in 2010 and 1266 kWh in 2018 [26].

3 Note that data on the temperature settings in residential buildings is not available either.

4 For instance, the percentage of free heat sources in the heat supply varies between 9 % and 21 % for the colder weather year 2010 and between 11 % and 29 % for the warmer weather year 2018, depending on the building type, at an indoor temperature of 21 °C. Note that solar radiation and hence solar gains are higher in 2018, which further decrease the external heating demand (refer to section 2.1).

## References

[1] Eurostat (2022). https://ec.europa.eu/eurostat/statistics-explained/index.php?title=Natural_gas_supply_statistics#Supply_structure.

[2] Destatis - Federal Statistical Office of Germany, Dashboard Deutschland (2022). https://www.dashboard-deutschland.de/energie/energie.

[3] Statista (2021). https://de.statista.com/statistik/daten/studie/151871/umfrage/erdgasbezug-deutschlands-aus-verschiedenen-laendern/.

[4] THEICE (2023). https://www.theice.com/products/27996665/Dutch-TTF-Gas-Futures/data.

[5] Uribe J.M., Mosquera-López S., Arenas O.J. (2022). Assessing the relationship between electricity and natural gas prices in European markets in times of distress. Energy Pol..

[6] Verivox (2023). https://www.verivox.de/gas/verbraucherpreisindex/.

[7] European Commission (2022). https://eur-lex.europa.eu/resource.html?uri=cellar:55edf05c-08d0-11ed-b11c-01aa75ed71a1.0001.02/DOC_1&format=PDF.

[8] European Commission, Council Regulation (Eu) (2023).

[9] Energiewende Agora (2022).

[10] Jülich Forschungszentrum (2022).

[11] (2022). BDEW - German Association of Energy and Water Industries, Kurzfristige Substitutions- und Einsparpotenziale Erdgas in Deutschland.

[12] Directorate-General for Energy, Kranzl L., Fallahnejad M., Büchele R., Müller A., Hummel M., Fleiter T., European Commission (2022).

[13] Praktiknjo A., Priesmann J., Kurzstudie (2022).

[14] Ruhnau O., Stiewe C., Muessel J., Hirth L. (2023). Natural gas savings in Germany during the 2022 energy crisis. Nat. Energy.

[15] Namazkhan M., Albers C., Steg L. (2020). A decision tree method for explaining household gas consumption: the role of building characteristics, socio-demographic variables, psychological factors and household behaviour. Renew. Sustain. Energy Rev..

[16] Brounen D., Kok N., Quigley J.M. (2012). Residential energy use and conservation: economics and demographics. Eur. Econ. Rev..

[17] Wyatt P. (2013). A dwelling-level investigation into the physical and socio-economic drivers of domestic energy consumption in England. Energy Pol..

[18] Sperber E., Frey U., Bertsch V. (2020). Reduced-order models for assessing demand response with heat pumps – insights from the German energy system. Energy Build..

[19] Harold J., Lyons S., Cullinan J. (2015). The determinants of residential gas demand in Ireland. Energy Econ..

[20] Elkhafif M.A. (1996). An iterative approach for weather-correcting energy consumption data. Energy Econ..

[21] (2022). Bmwk - Federal Ministry for Economic Affairs and Climate Action, Zahlen und Fakten: energiedaten: Nationale und internationale Entwicklung. Fassung vom 20.01.

[22] (2022). BDEW - German Association of Energy and Water Industries, Beheizungsstruktur des Wohnungsbestandes in Deutschland 2021.

[23] Klein S., Beckman A., Mitchell W., Duffie A. (2011).

[24] Loga T., Stein B., Diefenbach N., Born R. (2015).

[25] Destatis - Federal Statistical Office of Germany (2022). https://www-genesis.destatis.de.

[26] Hersbach H., Bell B., Berrisford P., Biavati G., Horányi A., Muñoz Sabater J., Nicolas J., Peubey C., Radu R., Rozum I., Schepers D., Simmons A., Soci C., Dee D., Thépaut J.-N. (2018).

[27] IWU - Institute for Housing and Environment (2022). https://www.iwu.de/publikationen/fachinformationen/energiebilanzen/#c205.

[28] Steinbach J. (2015).

[29] Greller M., Schröder F., Hundt V., Mundry B., Papert O. (2010). Universelle Energiekennzahlen für Deutschland - teil 2: verbrauchskennzahlentwicklung nach Baualtersklassen. Bauphysik.

[30] (2018). Destatis - Federal Statistical Office of Germany, Wohnen in Deutschland.

[31] Juhrich K. (2016).

[32] Destatis - Federal Statistical Office of Germany (2022).

[33] aktuell Gaspreis, NDR (2022). https://www.ndr.de/ratgeber/verbraucher/Gaspreis-aktuell-So-viel-kostet-Kilowattstunde,gaspreis142.html.

[34] Fleiter T., Elsland R., Rehfeldt M., Steinbach J., Reiter U., Catenazzi G., Jakob M., Rutten C., Harmsen R., Dittmann F., Riviere P., Stabat P. (2017).

[35] Ruhnau O., Muessel J. (2022).

[36] (2022). Destatis - Federal Statistical Office of Germany, Konsumausgaben und Lebenshaltungskosten: Private Konsumausgaben (Lebenshaltungskosten) nach der Haushaltsgröße.

[37] Schumacher K., Nissen C., Braungardt S. (2022).

[38] Streimikiene D., Lekavičius V., Baležentis T., Kyriakopoulos G.L., Abrhám J. (2020). Climate change mitigation policies targeting households and addressing energy poverty in European union. Energies.

[39] Labandeira X., Labeaga J.M., López-Otero X. (2017). A meta-analysis on the price elasticity of energy demand. Energy Pol..

[40] (2022). BMWK - Federal Ministry for Economic Affairs and Climate Action, Sicher durch den Winter: Zwischenbericht der ExpertInnen-Kommission Gas und Wärme.

[41] Republic of Austria - Parliament (2022). https://www.parlament.gv.at/PAKT/PR/JAHR_2022/PK1189/index.shtml#.

[42] Weber I., Wolff A. (2018). Energy efficiency retrofits in the residential sector – analysing tenants' cost burden in a German field study. Energy Pol..

[43] Grossmann K. (2019). Using conflicts to uncover injustices in energy transitions: the case of social impacts of energy efficiency policies in the housing sector in Germany. Global Transitions.

[44] Galvin R. (2015). The rebound effect, gender and social justice: a case study in Germany. Energy Pol..

[45] Schlör H., Fischer W., Hake J.-F. (2013). Sustainable development, justice and the Atkinson index: measuring the distributional effects of the German energy transition. Appl. Energy.

[46] Tirado Herrero S., Ürge-Vorsatz D. (2012). Trapped in the heat: a post-communist type of fuel poverty, {E}nergy. Policy.

[47] von Platten J. (2022). Energy poverty in Sweden: using flexibility capital to describe household vulnerability to rising energy prices. Energy Research \& Social Science.

[48] Streimikiene D., Kyriakopoulos G.L. (2023). Energy poverty and low carbon energy transition. Energies.

[49] Cischinsky H., Diefenbach N. (2018).

[50] Xiong J., Lian Z., Zhou X., You J., Lin Y. (2015). Effects of temperature steps on human health and thermal comfort. Building and Environment.

[51] Peeters L., de Dear R., Hensen J., D’haeseleer W. (2009). Thermal comfort in residential buildings: comfort values and scales for building energy simulation. Applied energy.

[52] Nielsen K.F., Holm G., Uttrup L.P., Nielsen P.A. (2004). Mould growth on building materials under low water activities. Influence of humidity and temperature on fungal growth and secondary metabolism. International Biodeterioration & Biodegradation.

